# The effect of gastrointestinal pathogen panel (GIP) on antibiotic management

**DOI:** 10.1017/ash.2021.162

**Published:** 2021-06-24

**Authors:** Christopher F. Saling, Maria T. Seville, Roberto L. Patron

**Affiliations:** 1 Division of Infectious Diseases, Mayo Clinic, Phoenix, Arizona; 2 Infectious Disease Providence Medical Group, Portland, Oregon

*To the Editor—*The gastrointestinal pathogen panel (GIP) offers detection via multiplexed polymerase chain reaction (PCR) for a multitude of bacterial, viral, and parasitic microbes associated with diarrheal illness. Its rapid turnaround time and high sensitivity has made GIP testing commonplace for the evaluation of diarrhea. Recent studies demonstrated its utility within the hospital setting. Torres-Miranda et al^1^ found that GIP testing led to decreased median hospital length of stay (LOS) by 4 days in patients with diarrhea. Furthermore, the mean time to appropriate antibiotic therapy was shortened by 24 hours in those who underwent GIP testing. In a retrospective Dutch study, Machiels et al^2^ concluded that if GIP had been implemented over conventional cultures, 83% of patients with diarrhea could have been removed from isolation precautions sooner. Axelrad et al^3^ conducted a retrospective study involving both inpatients and outpatients with diarrhea and reported that those who underwent GIP testing were less likely to be prescribed antibiotics and to undergo abdominal imaging or endoscopy. The utility of GIP in immunocompromised patients has also been documented. In patients with inflammatory bowel disease (IBD), Hong et al^4^ showed that GIP testing was associated with increased detection of microbes and lower rates of IBD therapy escalation and endoscopies with no difference in adverse outcomes. GIP testing in hematopoietic stem-cell transplant recipients has been associated with increased identification of infectious pathogens without increasing overall testing cost.^5^

However, despite the aforementioned potential benefits surrounding GIP testing, its high sensitivity to detect pathogens poses significant challenges to proper antibiotic stewardship practices. Also, for most patients, many of the microbes detected via GIP do not warrant antimicrobial treatment, potentially making the test economically wasteful. We examined the impact of GIP on antimicrobial management at our own institution. We conducted a retrospective study of 50 randomly selected patients hospitalized at Mayo Clinic in Arizona who were tested with a BioFire FilmArray GI PCR panel (bioMèrieux, Marcy-l'Étoile, France) between July and December 2019. Medical records were reviewed to capture gender, age, immunocompromised state, antibiotic use within 30 days, prior hospitalization within 3 months, history of *Clostridioides difficile* infection, time from admission to testing and GIP results. The primary endpoint of our study was to determine whether GIP results directly contributed to antibiotic management. This study was exempt from institutional review board approval.

In total, 26 patients were male and 24 were female; the average age was 61.7 years. Among them, 34 patients (68%) were immunocompromised. Overall, 41 GIP tests were ordered within 24 hours of admission. Of these, 22 patients (44%) had a positive GIP result and 5 were positive for 2 concurrent organisms. *C. difficile* was the most detected organism; it was identified in 16 (66.7%) of 24 positive tests. Overall, 11 patients (68.8%) with *C. difficile* had a recent hospitalization, antibiotics within 30 days, or a history of *C. difficile* infection. There were 3 cases of enteropathogenic *Escherichia coli*, 2 cases of enterotoxigenic *Escherichia coli*, 2 cases of adenovirus, 2 cases of norovirus, 1 case of rotavirus, and 1 case of *Vibrio cholerae*. Excluding *C. difficile*–positive patients, GIP testing changed the antibiotic management in 3 (6%) of 50 patients tested. One patient had antibiotics stopped appropriately; 1 patient received appropriate antibiotics, and 1 patient received inappropriate antimicrobial therapy.

Our results show that, except in the setting of *C. difficile* infection, GIP had little utility in guiding antimicrobial management. This was also true in the immunocompromised patient population. Other studies have similarly shown that *C. difficile* represented most pathogens found on GIP.^6^ Thus, it may be more efficient to first test patients hospitalized with diarrhea for *C. difficile* alone, especially in those with known risk factors for *C. difficile* infection. Furthermore, GIP testing does not differentiate between *C. difficile* colonization and active disease; therefore, it can lead to overtreatment for *C. difficile*. Although the GIP panel has many potential advantages compared to traditional testing with culture and ova and parasite examination, interpreting its results can prove difficult. Infectious disease consultants and antimicrobial and diagnostic stewardship teams should be involved to help guide the appropriate use of GIP testing.
